# Development and validation of the nomogram based on INR and eGFR for estimation of mortality in patients with acute-on-chronic hepatitis B liver failure

**DOI:** 10.1186/s12876-021-02054-3

**Published:** 2021-12-15

**Authors:** Shengnan Li, Xiehua Zhang, Qian Li, Binyue Lv, Yefan Zhang, Jianwei Jia, Xiaofen Yue, Wei Lu

**Affiliations:** 1 Tianjin Medical University Cancer Institute and Hospital, National Clinical Research Center for Cancer, Key Laboratory of Cancer Prevention and Therapy, Tianjin’s Clinical Research Center for Cancer, Tianjin, 300060 China; 2 Department of Hepatology, Tianjin Second People’s Hospital, Tianjin Institute of Hepatology, Tianjin, China; 3 Department of Infectious Diseases, The First Affiliated Hospital of Baotou Medical College, Baotou, Neimenggu China

## Abstract

**Aims and objectives:**

Acute-on-chronic hepatitis B liver failure (ACHBLF) is a critical clinical syndrome with a high short-term mortality evolved from chronic hepatitis B (CHB)-related liver disease. Prediction of mortality risk and early intervention can improve the prognosis of patients. This study aimed to develop and validate the nomogram for short-time mortality estimation in ACHBLF patients defined according to Asian Pacific Association for the Study of the Liver (APASL).

**Methods:**

A study of 105 ACHBLF patients with 90-day follow up was performed to develop the nomogram. Patients were randomly assigned to derivation cohort (n = 75) and validation cohort (n = 35) according to 7:3. Concordance index (C-index), calibration curve and decision curve analysis (DCA) were used to evaluate the nomogram. We also compared the nomogram with APASL ACLF research consortium (AARC) score, model for end-stage liver disease (MELD) score, MELD with serum sodium (MELD-Na) score and albumin-bilirubin (ALBI) score. The nomogram was validated using an external cohort including 40 patients.

**Results:**

The 28-day and 90-day mortality of 105 patients were respectively 49.52% and 55.24%. Albumin (ALB), international normalized ratio (INR) and estimated glomerular filtration rate (eGFR) were independent predictors for 28-day mortality; INR and eGFR were independent predictors for 90-day mortality. C-index of Nomogram-1 for 28-day mortality and Nomogram-2 for 90-day mortality were respectively 0.82 and 0.81. Calibration curve and Hosmer–Lemeshow test (Nomogram-1, 0.323; Nomogram-2, 0.231) showed optimal agreement between observed and predicted death. Areas under receiver operator characteristic curve(AUROC) of Nomogram-1(0.772) and Nomogram-2(0.771) were larger compared with AARC, MELD, MELD-Na and ALBI score. The results were well estimated in the external validation cohort.

**Conclusions:**

This study highlighted the predictive value of eGFR, and the nomogram based on INR and eGFR could effectively estimate individualized risk for short-term mortality of ACHBLF patients defined according to APASL.

**Supplementary Information:**

The online version contains supplementary material available at 10.1186/s12876-021-02054-3.

## Introduction

Acute-on-chronic liver failure (ACLF) is a critical clinical syndrome with acute decompensation of liver function based on chronic liver disease, characterized by systemic inflammation and high short-term mortality [1–3]. It can be accompanied by multi-organ dysfunction and progressed very rapidly. The main causes of ACLF are diverse between East and West. In China, acute-on-chronic hepatitis B liver failure (ACHBLF) accounts for more than 80% of all causes [4]. Thus, it is necessary to stratify ACHBLF patients for clinical decision- making depending on prognosis.

Until now model for end-stage liver disease (MELD) score and MELD with serum sodium (MELD-Na) score were most widely used in clinical to evaluate prognosis of patients with severe liver disease, however, with limited prognostic value. Recently APASL ACLF research consortium (AARC) derived and internally validated AARC score, which enrolled total bilirubin (TBIL), hepatic encephalopathy (HE) grades, international normalized ratio (INR), lactate and creatinine (Cr), showed fairly good predictive power [5]. However, AARC score is more complex and enrolls subjective factors, such as HE grades, which brings inconvenience to the wide promotion of clinical practice. Both AARC score and MELD score took into consideration extrahepatic organ failure, such as acute renal injury(AKI), and enrolled Cr as an evaluation index. However, Cr was easily affected by external factors and couldn't accurately represent AKI, so we aimed to enroll eGFR as a predictor, which could evaluate AKI more accurately.

In recent years, a variety of prognostic indices and models have been established with technological improvements in the field of molecular biology, genomics and transcriptomics, such as Neutrophil–lymphocyte ratio (NLR) [6], Th17/Treg [7], nomogram [8] and so on. Among them, nomogram has been proved to provide an individualized and highly accurate risk assessment [9, 10]. In this study, we aimed to develop and validate a nomogram to predict 28-day and 90-day mortality of ACHBLF patients defined according to Asian Pacific Association for the Study of the Liver (APASL). In addition, albumin-bilirubin (ALBI) score, which used to assess the severity of liver dysfunction in hepatocellular carcinoma (HCC) patients, was developed to evaluate the prognosis of ACLF patients lately [11–13]. In this study, we also aimed to estimate the prognostic value of ALBI score for ACHBLF patients.

## Materials and methods

### Study design and subjects

A cohort of ACHBLF patients who had been hospitalized in Tianjin Second People’s Hospital between January 2015 and December 2020 was enrolled. We developed a prognostic nomogram based on the above cohort, which was randomly assigned to derivation cohort (n = 75) and validation cohort (n = 35) according to 7:3 as described in previous studies [8]. Nomogram discrimination was assessed by concordance index (C-index), and calibration was conducted with calibration curve and Hosmer–Lemeshow test. Decision curve analysis (DCA) was proposed to assess the clinical validity of the predictive model. The predictive value between the nomogram with AARC, MELD, MELD-Na and ALBI score was performed by receiver operating characteristic (ROC) curve. In addition, we used an external cohort with identical characteristics for validation.

Clinical data was collected from medical records within 24 h of established diagnosis, including age, gender, pathologic basis, total bilirubin (TBIL), albumin(ALB), cholinesterase (CHE), Cr, blood urea nitrogen (BUN), estimated glomerular filtration rate (eGFR), international normalized ratio (INR), serum sodium (Na), lactate, HBV-DNA, mean arterial pressure(MAP), hepatic encephalopathy (HE), ascite and so on. All patients were followed up by telephone and lasted for 28 days, then 90 days if survived at 28 days. The outcome was recorded as survival or death. Patients who underwent liver transplantation from medical records were excluded. This study was approved by the Ethics Committee of the Tianjin Second People’s Hospital (018-18). Informed consent was waived by Ethics Committee of the Tianjin Second People’s Hospital due to the study’s retrospective nature.

### Inclusion and exclusion criteria

All patients enrolled were diagnosed according to ACLF criteria of Asian Pacific Association for the Study of the Liver (APASL) [14]. ACLF was defined as acute decompensation of liver function, presented as severe gastrointestinal symptoms, hyperbilirubinemia and abnormal blood coagulation coagulopathy, with or without hepatic encephalopathy, which was developed from chronic hepatitis B (CHB) characterized by serum hepatitis B surface positive ≥ 6 months and histological evidence of chronic hepatitis. Exclusion criteria included: (1) coinfection with other hepatitis viruses or human immunodeficiency virus; (2) acute, subacute and chronic liver failure; (3) liver failure caused by other factors, such as autoimmune hepatitis, alcoholic liver disease and drug-induced hepatitis; (4) received liver transplantation; (5) renal dysfunction caused by underlying renal disease; (6) complicated with liver cancer, other malignant tumor or severe diseases in other organs.

### Statistical analysis

Continuous variables were expressed as mean ± standard deviation, and categorical variables were represented as absolute and relative frequencies. Differences in continuous variables were analyzed using Student’s *t* test or Mann–Whitney *U* test, while Chi-square test or Fisher’s exact test was used for categorical variables. Logistic regression analysis was used to identify the independent prognostic predictors. Variables with *P* < 0.20 in the univariate logistic regression analysis were progressed to a multivariate analysis using stepwise regressions. All statistical tests were two-sided, and *P* < 0.05 were considered to be statistically significant. Odds ratio (OR) and 95% confidence interval (CI) were calculated. Nomogram was developed via *rms* package in R version 4.0.2.

### Formulas

- MELD score [15] = 3.8 × ln (TBIL mg/dL) + 9.6 × ln (Creatinine mg/dL) + 11.2 × ln (INR) + 6.4;
- MELD-Na score [16] = MELD + 1.59 × (135 − Na);
- ALBI score [12] = − 0.085 × (Albumin g/L) + 0.66 × log10 (TBIL µmol/L);
- CKD-EPI eGFR [17] = 141×min (Cr/κ, 1)^α^×max(Cr/κ,1)^−1.209^×0.993^Age^×1.018 (if female)×1.159 (if black);
- AARC score was referenced according to [5].

## Results

### Patient characteristics

**Fig. 1:**
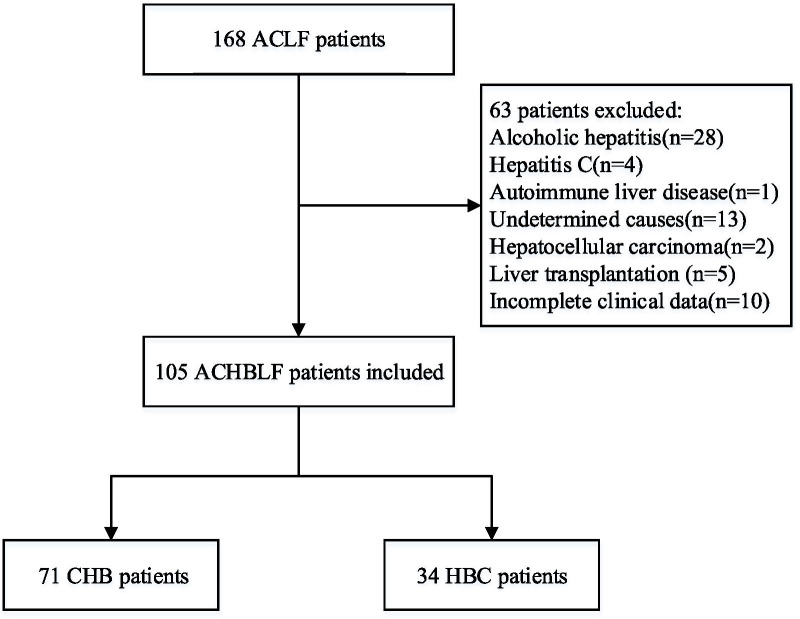
Flowchart for patient inclusion. ACLF, acute-on-chronic liver failure; ACHBLF, acute-on-chronic hepatitis B liver failure; CHB, chronic hepatitis B; HBC, hepatitis B cirrhosis

We collected clinical data of 168 ACLF patients, and 105 patients were finally enrolled in accordance with the criteria (Fig. 1). The 105 patients comprised 80 men and 25 women, aged 23–74 years old (47.87 ± 12.07 years old), including 71 CHB (67.62%) and 34 hepatitis B cirrhosis (HBC) (32.38%) patients. The baseline characteristics were no statistical difference between derivation cohort and validation cohort (Table 1).

**Table 1:** Baseline characteristics of the derivation cohort and validation cohort

Variables	Derivation cohort (n = 75)	Validation cohort (n = 30)	*P*
Gender (%)			
Male/female	19/56	6/24	0.740
Age	49.03 ± 11.70	44.97 ± 12.68	0.140
Pathologic basis (%)			
CHB	53 (70.67%)	18 (60%)	
HBC	22 (29.33%)	12 (40%)	0.410
ALB (g/L)	31.61 ± 5.13	31.46 ± 5.35	0.930
TBIL (umol/L)	314.38 ± 104.00	328.73 ± 147.91	0.980
Na (mmol/L)	136.48 ± 3.94	135.01 ± 4.59	0.140
Cr (umol/L)	84.89 ± 61.13	80.05 ± 33.36	0.530
BUN (mmol/L)	6.33 ± 5.40	5.86 ± 2.59	0.480
CHE (U/L)	4012.60 ± 1506.31	3936.47 ± 1735.93	0.800
eGFR (ml/min)	98.03 ± 30.37	100.57 ± 32.53	0.720
INR	2.53 ± 1.31	2.72 ± 0.98	0.098
Lactate	2.10 ± 1.58	2.30 ± 1.26	0.260
MAP	84.11 ± 10.70	85.90 ± 10.11	0.260
HBV-DNA	7.70E + 04 (2.04E + 03,6.75E + 07)	2.39E + 05 (2.63E + 04,1.08E + 08)	0.120
HE			
Yes/no	16/59	7/23	1.000
Ascite			
Yes/no	71/4	30/0	0.470
AKI			
Yes/no	13/62	7/23	0.479
MELD score	24.81 ± 6.68	26.10 ± 5.68	0.355
AARC score	8.68 ± 1.85	9.27 ± 1.82	0.143
ALBI score	− 1.04 ± 0.45	− 1.04 ± 0.44	0.830

CHB, chronic hepatitis B; HBC, hepatitis B cirrhosis; ALB, albumin; TBIL, total bilirubin; Na, serum sodium; Cr, creatinine; BUN, blood urea nitrogen; CHE, cholinesterase; eGFR, estimated glomerular filtration rate; INR, international normalized ratio; MAP, mean arterial pressure; HE, hepatic encephalopathy; AKI, acute renal injury; MELD, model for end-stage liver disease; AARC, APASL ACLF research consortium; ALBI, albumin-bilirubin

The overall 28-day mortality was 49.52% (52/105), and 90-day mortality was 55.24% (58/105). In 71 CHB patients, 28-day mortality was 53.52% (38/71) and 90-day mortality was 60.56% (43/71); In 34 HBC patients, 28-day mortality was 41.18% (14/34) and 90-day mortality was 44.12% (15/34), with statistically significant difference (*P* = 0.000). Age, pathologic basis, ALB, CHE, eGFR, INR, and HE were independent risk predictors by univariate logistic regression analysis. ALB, eGFR, INR were independent risk predictors for 28-day mortality, while eGFR and INR as independent risk predictors for 90-day mortality by multivariate logistic regression analysis. The results were showed in Table 2.

**Table 2:** Univariate and Multivariate logistic regression analysis of mortality risk

Characteristics	28-Day			90-Day		
	*P*u	*P*m	OR (95% CI)	*P*u	*P*m	OR (95% CI)
Gender	0.74			0.38		
Age	0.01	0.143	1.052 (0.984–1.130)	0.02		
Pathologic basis	0.15			0.11	0.090	0.301 (0.066–1.129)
ALB (g/L)	0.03	0.008	0.706 (0.527–0.888)	0.06		
TBIL(µmol/L)	0.63			0.54		
Cr (µmol/L)	0.06			0.06		
BUN (mmol/L)	0.10			0.04		
CHE (U/L)	0.02	0.057	1.001 (1.000–1.002)	0.03		
eGFR (ml/min)	0.00	0.031	0.962 (0.923–0.993)	0.01	0.021	0.974 (0.951–0.995)
INR	0.00	0.010	3.872 (1.565–12.395)	0.00	0.020	2.226 (1.195–4.767)
Lactate	0.04			0.12		
MAP	0.20			0.22		
HBV-DNA	0.41			0.37		
HE	0.00	0.054	16.717 (1.480–569.488)	0.01	0.055	9.194 (1.337–190.739)
Ascite	0.32			0.69		
ALBI score	0.04			0.07		

ALB, albumin; TBIL, total bilirubin; Cr, creatinine; BUN, blood urea nitrogen; CHE, cholinesterase; eGFR, estimated glomerular filtration rate; INR, international normalized ratio; MAP, mean arterial pressure; HE, hepatic encephalopathy; ALBI, albumin-bilirubin; *P*u, *p* value of univariate logistic regression analysis; *P*m, *p* value of multivariate logistic regression analysis

### Development and validation of the prognostic nomogram

**Fig. 2:**
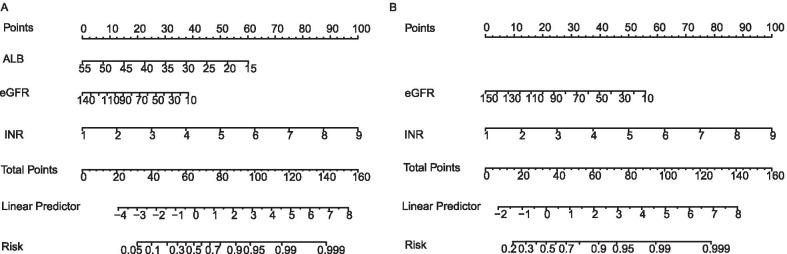
Prognostic nomograms of the training cohort. **A** Prognostic nomogram for 28-day mortality. **B** Prognostic nomogram for 90-day mortality. To use the nomogram, a line is drawn upward to determine the number of points received for each variable value. The sum of these scores is located on the total points axis and draw a line straight down to determine the probability of mortality. eGFR, estimated glomerular filtration rate; INR, international normalized ratio

Two prognostic nomograms were developed incorporating independent risk predictors for 28-day mortality (Nomogram-1) and 90-day mortality (Nomogram-2). By calculating the total points through a vertical line from the variable to the point axis, we could estimate the probability of short-time mortality. The nomograms were showed in Fig. 2.

**Fig. 3:**
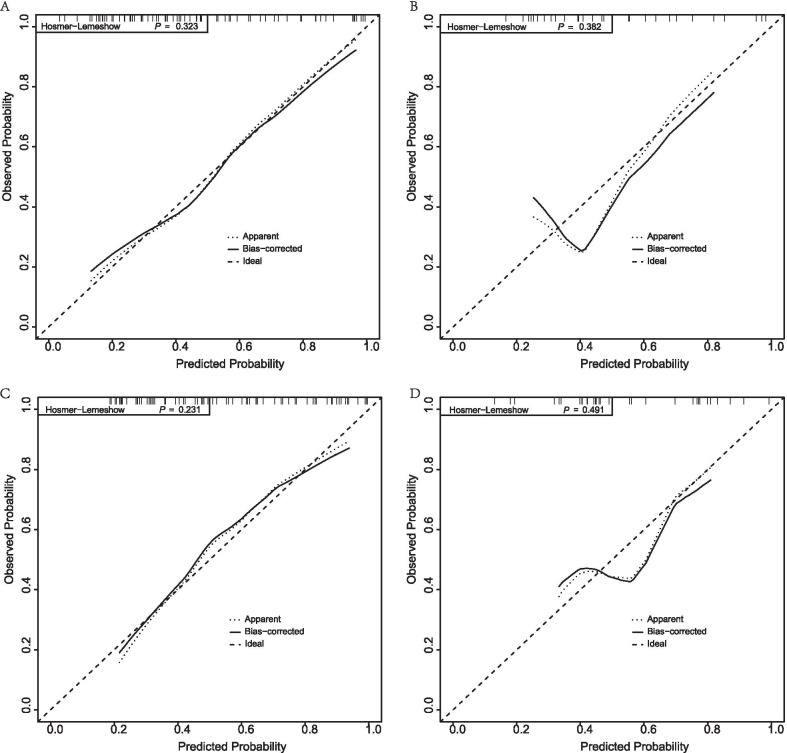
The calibration curves of the nomogram for 28-day and 90-day mortality in the training cohort and validation cohort. **A** The calibration curve in the training cohort at 28-day. **B** The calibration curve in the validation cohort at 28-day. **C** The calibration curve in the training cohort at 90-day. **D** The calibration curve in the validation cohort at 90-day

**Fig. 4:**
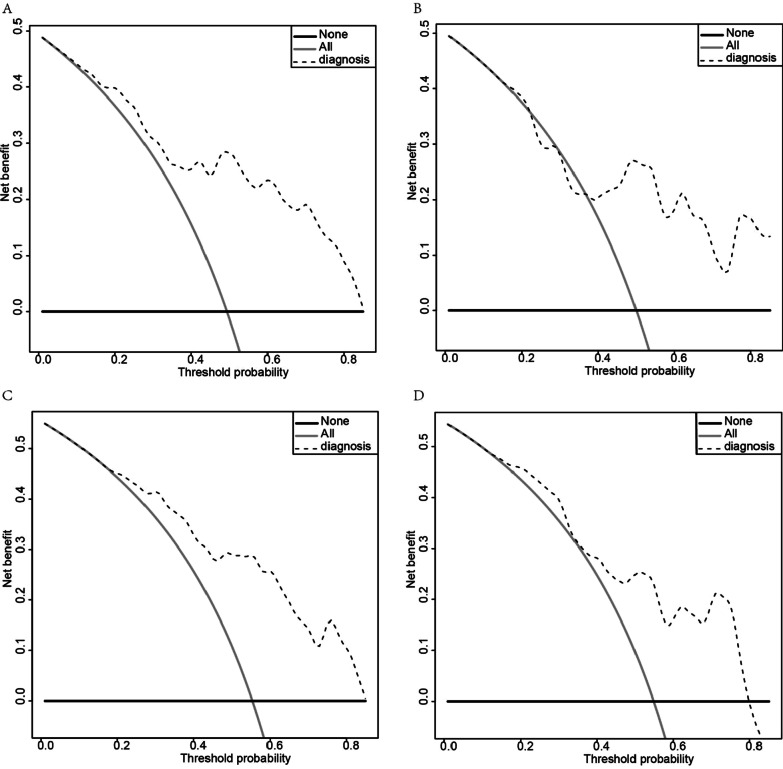
Decision curve analysis of the nomogram for 28-day and 90-day mortality in the training cohort and validation cohort. **A** Decision curve analysis in the training cohort at 28-day. **B** Decision curve analysis in the validation cohort at 28-day. **C** Decision curve analysis in the training cohort at 90-day. **D** Decision curve analysis in the validation cohort at 90-day

In derivation cohort, C-index of Nomogram-1 was 0.82 (95% CI 0.72–0.91) and C-index of Nomogram-2 was 0.81 (95% CI 0.71–0.91), which revealed excellent discriminative abilities (Table 3). Calibration curve and Hosmer–Lemeshow test (Nomogram-1, 0.323; Nomogram-2, 0.231) indicated excellent calibration between nomogram predictions and actual observations. In validation cohort, C-index also showed good discriminative abilities with 0.73 (95% CI 0.54–0.92) for Nomogram-1 and 0.74 (95% CI 0.56–0.91) for Nomogram-2. Calibration curve and Hosmer–Lemeshow test (Nomogram-1, 0.382; Nomogram-2, 0.491) also indicated a good calibration. DCA confirmed the improved clinical utility of the nomogram in predicting mortality of ACHBLF patients both in derivation cohort and validation cohort. Calibration curve and DCA were showed in Figs. 3, 4. When subjected to the external validation, C-index was 0.78(95% CI 0.70–0.87) for Nomogram-1 and 0.88(95% CI 0.75–1.02) for Nomogram-2. Hosmer–Lemeshow test for Nomogram-1and Nomogram-2 were 0.362 and 0.178. C-index and calibration curve showed excellent discrimination and calibration ability. DCA confirmed the improvement of the nomogram on clinical decision-making. DCA of the external cohort were showed in Additional file 1: Figure S1. C-index and Hosmer–Lemeshow test were also showed in Table 3.

**Table 3:** C-index and Hosmer–Lemeshow test of the derivation, validation and external cohort

	28-Day			90-Day		
	Derivation (95% CI)	Validation (95% CI)	External (95% CI)	Derivation (95% CI)	Validation (95% CI)	External (95% CI)
C-index	0.82 (0.72–0.91)	0.73 (0.54–0.92)	0.78 (0.70–0.87)	0.81 (0.71–0.91)	0.74 (0.56–0.91)	0.88 (0.75–1.02)
H–L test	0.323	0.382	0.362	0.231	0.491	0.178

CI, confidence interval; H–L, Hosmer–Lemeshow

### Comparison of predictive value between nomograms and clinical predictive models

The AUROC of Nomogram-1and Nomogram-2 for 28-day mortality were respectively 0.772 and 0.771, which showed similar predictive accuracy (Additional file 2: Figure S2).

**Fig. 5:**
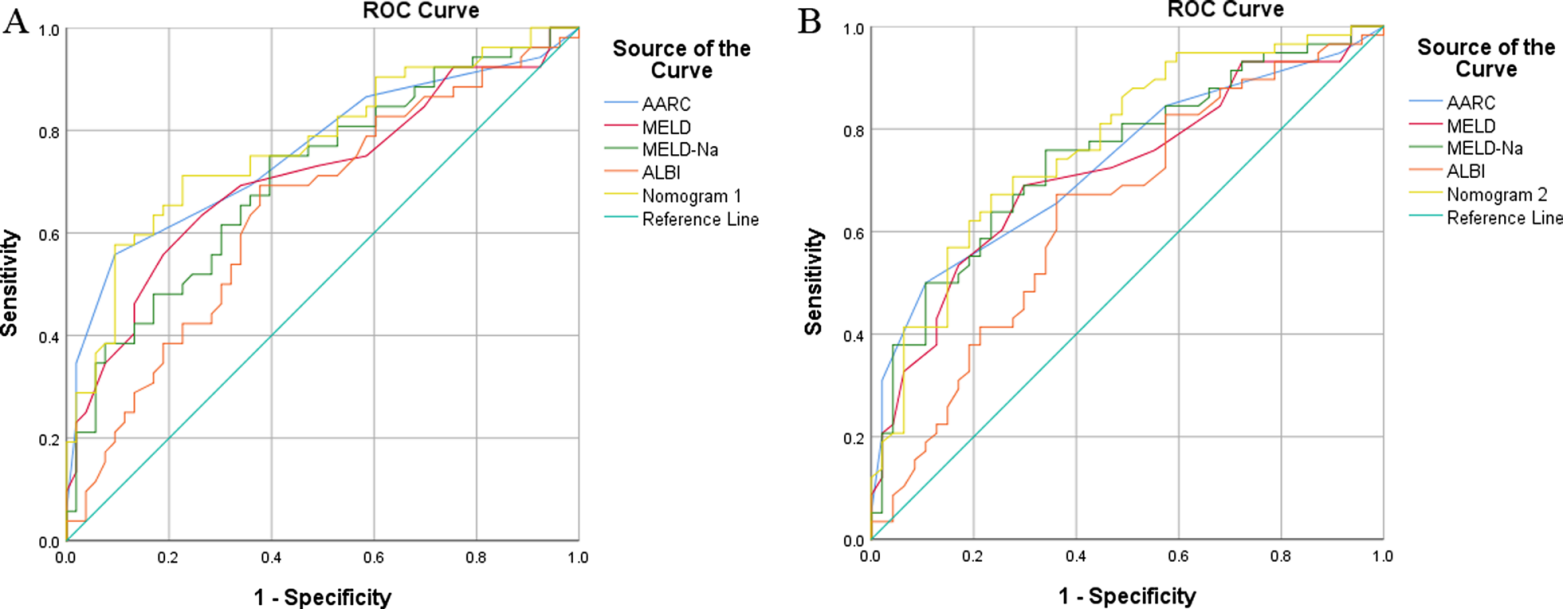
Receiver operating characteristic curves of the nomogram, AARC, MELD, MELD-Na and ALBI score for predicting mortality of ACHBLF. **A** Receiver operating characteristic curves of Nomogram 1, AARC, MELD, MELD-Na and ALBI score. **B** Receiver operating characteristic curves of Nomogram 2, AARC, MELD, MELD-Na and ALBI score.

AUROC of Nomogram-1 (0.772) was larger than AARC (0.759), MELD (0.712), MELD-Na (0.710) and ALBI (0.646) score. AUROC of Nomogram-2 (0.771) was also larger than AARC (0.732), MELD (0.718), MELD-Na (0.746) and ALBI (0.641) score (Fig. 5). The two nomograms both showed superior predictive accuracy.

## Discussion

ACLF is one of the most challenging health problems worldwide, characterized by acute onset, rapid emergence and extremely high short-term mortality [18, 19]. Prediction of mortality risk and early intervention can improve the prognosis of patients. Nowadays, the development of nomogram allows clinicians to standardize clinical decision through an evidence-based and fully-individualized tool. However, as we know, there was few study on nomogram specifically for ACHBLF patients. In this study, we developed and validated a nomogram based on INR and eGFR for short-term mortality prediction in ACHBLF patients defined according to APASL. Internal and external validations showed the nomogram with relatively high C-index and well-fitted calibration curves, which estimated the reliability and generalizability of the nomogram. Additionally, the performance of nomograms was, in turn, validated by ROC curve which showed better predictive value than AARC, MELD, MELD-Na and ALBI score.

In terms of clinical indicators, independent predictors for short-time mortality were ALB, INR and eGFR. ALB was included in Child–Pugh score to evaluate liver function, and lower ALB might represent poor liver function, which tended to aggravate disease progress. Consistent with previous studies [20, 21], INR, as the most commonly used index for coagulation assessment, was proved to be a high risk factor for prognosis of ACHBLF patients in our study. It was important to stress that, eGFR, but not Cr, was a powerful predictor of mortality in our data, which probably estimated its better prognostic value than Cr [22, 23]. Different with previous studies [5, 24], TBIL, MAP, lactate and HBV-DNA were not associated with short-time mortality in ACHBLF patients, which probably could be explained by the homogeneity of study population. In addition, HE, a well-established prognostic factor in AARC score, was also not identified as a prognostic factor in our data, but with a marginal statistical difference. Moreover, regardless of cirrhosis, ACLF patients developed from CHB also had a high mortality rate, and there was no significant difference between non-cirrhotic and cirrhotic HBV populations in short-time mortality, which further estimated the viewpoint of World Gastroenterology Organization [25].

Generally speaking, the nomogram would improve prognostic capabilities for mortality of ACHBLF patients. Parameters in the nomogram could be easily obtained and it would provide a user-friendly interface without computer software. More importantly, we highlighted the predictive value of eGFR. Nevertheless, our study had two main limitations. Firstly, the nomogram was developed based on a retrospective study from a single center, which might not represent the entire locally ACHBLF patients. Secondly, the nomogram was developed based on a relatively small group of patients, and was validated in only one external cohort, so a prospective multicenter clinical research might be needed to further improve and validate the nomogram.

## Conclusions

In conclusion, we developed and validated a nomogram based on INR and eGFR for short-time mortality estimation in ACHBLF patients defined according to APASL. This study highlighted the predictive value of eGFR and could strengthen prognosis-based decision-making.

## Supplementary Information

**Additional file 1. Figure. S1** Decision curve analysis of the external cohort at 28-day and 90-day. **A** Decision curve analysis at 28-day. **B** Decision curve analysis at 90-day.

**Additional file 2.** **Figure. S2** Receiver operating characteristic curves of Nomogram 1 and Nomogram 2.

## Data Availability

All data generated or analyzed during this study are included in this article.

## Abbreviations

ACHBLF: Acute-on-chronic hepatitis B liver failure
CHB: Chronic hepatitis B
C-index: Concordance index
DCA: Decision curve analysis
ALBI: Albumin-bilirubin
ROC: Receiver operating characteristics curve
ACLF: Acute-on-chronic liver failure
AARC: APASL ACLF research consortium
MELD: Model for end-stage liver disease
MELD-Na: MELD with serum sodium
NLR: Neutrophil–lymphocyte ratio
HCC: Hepatocellular carcinoma
APASL: Asian Pacific Association for the Study of the Liver
OR: Odds ratio
CI: Confidence interval
HBC: Hepatitis B cirrhosis
TBIL: Total bilirubin
ALB: Albumin
Na: Serum sodium
Cr: Creatinine
BUN: Blood urea nitrogen
CHE: Cholinesterase
eGFR: Estimated glomerular filtration rate
INR: International normalized ratio
MAP: Mean arterial pressure
HE: Hepatic encephalopathy
