# Supplementary material for: Development and validation of the nomogram based on INR and eGFR for estimation of mortality in patients with acute-on-chronic hepatitis B liver failure
Source: BMC Gastroenterol. 2021 Dec 15;21:474. doi: 10.1186/s12876-021-02054-3 (PMC8675499; doi:10.1186/s12876-021-02054-3)
Supplement: Supplementary file 1 — Additional file 1. Figure. S1 Decision curve analysis of the external cohort at 28-day and 90-day. A Decision curve analysis at 28-day. B Decision curve analysis at 90-day. [file 12876_2021_2054_MOESM1_ESM.pdf]

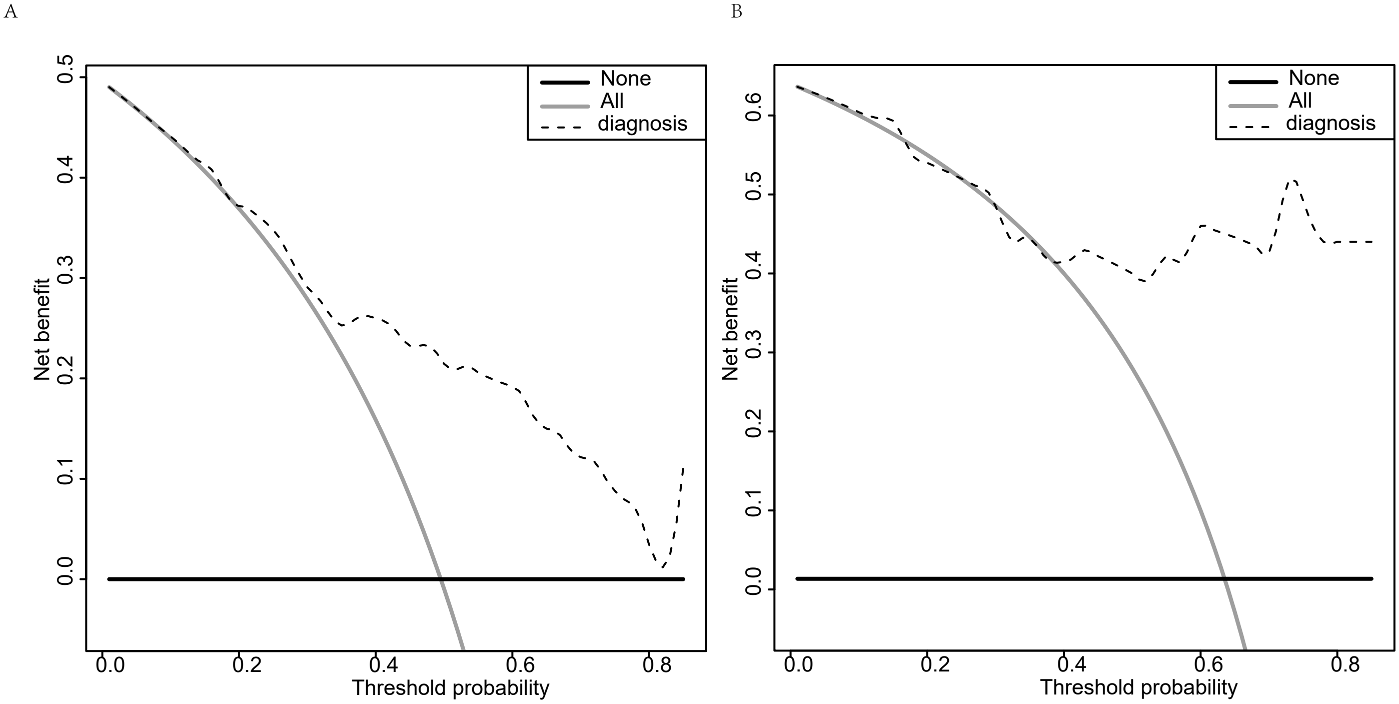

Figure.S1 Decision curve analysis of the external cohort at 28-day and 90-day. (A) Decision curve analysis at 28-day. (B) Decision curve analysis at 90-day.
