# Supplementary figures and images for: Development and validation of the nomogram based on INR and eGFR for estimation of mortality in patients with acute-on-chronic hepatitis B liver failure
Source: BMC Gastroenterol. 2021 Dec 15;21:474. doi: 10.1186/s12876-021-02054-3 (PMC8675499; doi:10.1186/s12876-021-02054-3)

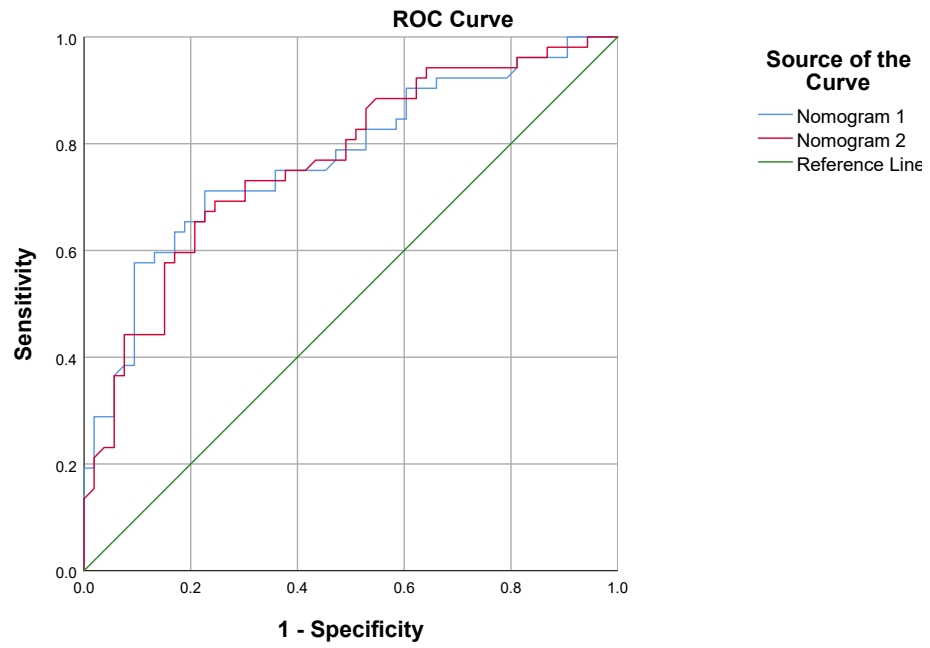

Figure.S2 Receiver operating characteristic curves of Nomogram 1 and Nomogram 2.

Supplement: Supplementary file 2 — Additional file 2. Figure. S2 Receiver operating characteristic curves of Nomogram 1 and Nomogram 2. [file 12876_2021_2054_MOESM2_ESM.pdf]
